# New Books

**Published:** 2008-06

**Adsorption of Metals by Geomedia II, 7**

Mark Barnett, Douglas Kent, eds.

St. Louis, MO:Elsevier, 2007. 430 pp. ISBN: 978-0-444-53212-1, $140

**Advances in Molecular Toxicology, Vol 2**

James C. Fishbein

St. Louis, MO:Elsevier, 2008. 275 pp. ISBN: 978-0-444-53098-1, $220

**Arctic–Subarctic Ocean Fluxes: Defining the Role of the Northern Seas in Climate**

Robert R. Dickson, Jens Meincke, Peter Rhines, eds.

New York:Springer, 2008, 738 pp. ISBN: 978-1-4020-6773, $219

**Biosecurity and Bioterrorism: Containing and Preventing Biological Threats**

Jeffrey Ryan, Jan Glarum

St. Louis, MO:Elsevier, 2008. 352 pp. ISBN: 978-0-7506-8489-7, $69.95

**Cleanup of Chemical and Explosive Munitions**

Richard Albright

St. Louis, MO:Elsevier, 2008. 330 pp. ISBN: 978-0-8155-1540-1, $125.00

**Comparative Toxicogenomics, 2**

Christer Hogstrand, Pete Kille

St. Louis, MO:Elsevier, 2008. 250 pp. ISBN: 978-0-444-53274-9, $110.00

**Economics of the Environment: Theory and Policy, 7th ed.**

Horst Siebert

New York:Springer, 2008. 333 pp. ISBN: 978-3-540-73706-3, $109

**Economics of Poverty, Environment and Natural-Resource Use**

Rob B. Dellink, Arjan Ruijs, eds.

New York:Springer, 2008. 218 pp. ISBN: 978-1-4020-8302-0, $119

**Energy… beyond Oil**

Fraser Armstrong, Katherine Blundell

New York: Oxford University Press, 2007. 240 pp. ISBN: 978-0-19-920996-5, $49.50

**Estimating Mortality Risk Reduction and Economic Benefits from Controlling Ozone Air Pollution**

Committee on Estimating Mortality Risk Reduction Benefits from Decreasing Tropospheric Ozone Exposure, National Research Council

Washington, DC:National Academies Press, 2008. 206 pp. ISBN: 978-0-309-11994-8, $45.75

**Food Contaminants and Residue Analysis, 51**

Yolanda Picó

St. Louis, MO:Elsevier, 2008. 672 pp. ISBN: 978-0-444-53019-6, $310

**Human Toxicology of Chemical Mixtures**

Harold Zeliger

St. Louis, MO:Elsevier, 2008. 400 pp. ISBN: 978-0-8155-1589-0, $249

**Impact of Pollution on Animal Products**

Bernard Faye, Yuriy Sinyavski, eds.

New York:Springer, 2008. 205 pp. ISBN: 978-1-4020-8357-0, $149.95

**Institutions and Environmental Change: Principal Findings, Applications, and Research Frontiers**

Oran R. Young, Leslie A. King, Heike Schroeder, eds.

Cambridge, MA:MIT Press, 2008. 400 pp. ISBN: 978-0-262-24057-4, $70

**Nuclear Risk in Central Asia**

Brit Salbu, Lindis Skipperud, eds.

New York:Springer, 2008. 237 pp. ISBN: 978-1-4020-8315-0, $149.95

**Persistent Organic Pollutants in Asia, 7**

An Li, Shinsuke Tanabe, Guibin Jian, John Giesy, Paul Lam, eds.

St. Louis, MO:Elsevier, 2008. 842 pp. ISBN: 978-0-08-045132-9, $185

**Sittig’s Handbook of Toxic and Hazardous Chemicals and Carcinogens, 5th ed.**

Richard Pohanish, ed.

St. Louis, MO:Elsevier, 2008. 3,000 pp. ISBN: 978-0-8155-1553-1, $595

**State of the Wild 2008–2009: A Global Portrait of Wildlife, Wildlands, and Oceans**

Wildlife Conservation Society

Washington, DC:Island Press, 2008. 312 pp. ISBN: 978-1-59726-135-7. $29.94

**Water Policy in Australia: The Impact of Change and Uncertainty**

Lin Crase

Washington, DC:RFF Press, 2008. 300 pp. ISBN: 978-1-933115-58-0, $70

**Water War in the Klamath Basin: Macho Law, Combat Biology, and Dirty Politics**

Holly Doremus, A. Dan Tarlock

Washington, DC:Island Press, 2008. 260 pp. ISBN: 978-1-59726-393-1, $60

